# Alterations in Regulatory T Cell Subpopulations Seen in Preterm Infants

**DOI:** 10.1371/journal.pone.0095867

**Published:** 2014-05-05

**Authors:** Angel A. Luciano, Ileana M. Arbona-Ramirez, Rene Ruiz, Braulio J. Llorens-Bonilla, Denise G. Martinez-Lopez, Nicholas Funderburg, Morna J. Dorsey

**Affiliations:** 1 Department of Pediatrics, Division of Neonatology, University of South Florida, Morsani College of Medicine, Tampa, Florida, United States of America; 2 Department of Microbial Infection and Immunity, The Ohio State University, Columbus, Ohio, United States of America; 3 Department of Pediatrics, Division of Allergy, Immunology and Bone Marrow Transplant, University of California San Francisco, San Francisco, California, United States of America; University of Giessen Lung Center, Germany

## Abstract

Regulatory T cells are a population of CD4+ T cells that play a critical role in peripheral tolerance and control of immune responses to pathogens. The purpose of this study was to measure the percentages of two different regulatory T cells subpopulations, identified by the presence or absence of CD31 (Recent thymic emigrants and peripherally induced naïve regulatory T cells), in term and preterm infant cord blood. We report the association of prenatal factors, intrauterine exposure to lipopolysaccharide and inflammation and the percentages of these regulatory T cell subpopulations in term and preterm infants. Cord blood samples were collected from both term and preterm infants and mononuclear cells isolated over a Ficoll-Hypaque cushion. Cells were then stained with fluorochrome-labeled antibodies to characterize regulatory T cell populations and analyzed with multi-color flow cytometry. Cord blood plasma C-reactive protein, and lipopolysaccharide were also measured. Placental pathology was also examined. We report a gestational age-dependent difference in the percentage of total regulatory T cells, in which preterm infants of lower gestational ages have an increased percentage of regulatory T cells. We report the presence of two populations of regulatory T cells (CD31+ and CD31-) in cord blood of term and preterm infants and their association with different maternal and fetal characteristics. Factors associated with differences in the percentage of CD31- Tregs included the use of prenatal antibiotics, steroids and magnesium sulfate. In addition, the percentage of CD31- Tregs was significantly higher in cord blood of preterm pregnancies associated with inflammation and prenatal lipopolysaccharide exposure. The peripheral Treg pool of preterm infants could be altered by prenatal exposure to inflammation and chorioamnionitis; however, the clinical implications of this finding are not yet understood.

## Introduction

Regulatory T cells (Tregs) are a population of CD4^+^ T cells that play a critical role in peripheral tolerance and control of immune responses to pathogens [1]. Studies of Tregs have identified different populations of cells with immunosuppressive capabilities but with different cell surface markers, site and mode of generation [2], [3]. Forkhead box P3 (FOXP3) positive T cells, the most extensively characterized Tregs with suppressive function, are often defined by the surrogate markers CD4 and CD25. Two origins have been described for FOXP3^+^ T cells – the thymus and the periphery. In the thymus, FOXP3^+^ T cells are generated by positive selection of conventional CD4^+^ T cells. In the periphery, a number of triggers induce the expression of FOXP3 in naïve CD4^+^ T cells [1].

There is evidence that T cells with a naïve CD4^+^CD25^+^CD45RA^+^ surface profile and immunosuppressive properties are detectable within the peripheral Treg pool [4], [5]. Unlike the antigen-primed Tregs, this naïve subset may comprise de novo generated cells that have been recently released from the thymus and have not yet experienced antigen exposure. Surface expression of CD31 on naïve CD4^+^ T cells distinguishes recent thymic emigrants (RTEs) from peripherally expanded naïve T cells (CD31^−^) [6]. RTEs, unlike the naïve T cells lacking CD31, contain high levels of T cell receptor (TCR) excision circles (TRECs). TRECs are generated as a by-product of the TCR rearrangement process in the thymus and are enriched in newly generated T cells [7].

The T cell immune response in preterm infants is assumed to be dysregulated and affected by prenatal factors including in utero inflammation and maternal characteristics [8]–[10]. However, little investigation has elucidated the role of prenatal factors and inflammation in Treg homeostasis. Treg mediated inhibition of antimicrobial immune responses could lead to ineffective clearance of pathogens resulting in chronic inflammation from persistent infection. On the other hand, Tregs participate in abrogating immune responses thereby preventing exacerbated and potentially deleterious immune activation [11]. The purpose of this study was to measure the percentages of two different Treg subpopulations (RTEs and peripherally induced naïve Tregs) in term and preterm infant cord blood. In addition, we report the association of prenatal factors, intrauterine exposure to lipopolysaccharide (LPS) and inflammation with the percentages of these Tregs subpopulation in term and preterm infants. We hypothesized that a shift in the homeostatic composition of Treg cell subsets related to reduced de novo generation of RTE Tregs is associated with prematurity and LPS burden. We performed flow cytometric analysis of the two different Treg populations in cord blood of term and preterm infants. We recently demonstrated that preterm infant cord blood has higher LPS levels than term infant cord blood [12] and in this study we sought to investigate the relationship between Treg populations and LPS in cord blood, chorioamnionitis, and both maternal and fetal factors.

The percentage of Tregs in neonates has been described by others [13], [14], but there is limited information about the distribution of Treg subpopulations, particularly in preterm infants. What is not known is the influence of maternal or fetal factors associated with prematurity on the development of RTE Tregs. To our knowledge this is the first study to examine RTE Tregs in our population of interest.

## Methods

### Subjects

Cord blood (CB) samples were collected from infants delivered at Tampa General Hospital (TGH) after obtaining written informed consent from mothers for this University of South Florida IRB approved study. These studies were performed in accordance with the policies of and approval of the Institutional Review Boards at the University of South Florida (USF) and TGH. Subjects included healthy term infants (gestational age ≥37 weeks, n = 31) and preterm infants (gestational age ≤36 weeks, n = 40). Demographic and clinical details for the infants and mothers were obtained from the medical records.

### Cord blood collection

CB was collected using sterile technique. After the placenta was delivered, a section of the umbilical cord was cleaned with povidone-iodine or alcohol topical antiseptics. The umbilical vein was identified and blood was obtained using a 21G needle. Blood was then collected into EDTA-coated tubes, and samples were transported to the laboratory.

### Cell preparation and flow cytometry

Cord blood mononuclear cells (CBMCs) were isolated within 24 hours of collection by using centrifugation over a Ficoll-Histopaque cushion and red blood cells lysed with Pharm Lyse (BD Biosciences, San Jose, CA, USA) for 10 minutes. Isolated CBMCs were resuspended in 10% dimethyl sulfoxide (DMSO) and 90% fetal bovine serum (FBS). CBMC aliquots were then stored at −80°C for 24 hours and then transferred to −150°C storage.

CBMCs were thawed; a cell viability test that used propidium iodide staining consistently yielded viability of ≥95%. CBMCs were stained with fluorochrome-labeled antibodies to characterize T-cell populations (**Table 1**). FOXP3 intranuclear staining (eBioscience, San Diego, CA, USA) was performed according to manufacturer's protocol following cell surface staining. For multi-color flow cytometry analysis, cells were first stained with surface antibodies specific for CD3, CD4, CD25, CD45RA, CD45RO and CD31 followed by intranuclear staining for FOXP3. Fluorescence Minus One (FMO) controls were used to identify any background spread of fluorochromes and to establish gating boundaries.

**Table 1:** Cell surface markers and fluorochrome labeling.

Markers	Fluorochromes, isotypes, clones and company
**CD3**	Pacific Blue, Ms IgG_1,k_, UCHT1, BD Biosciences
**CD4**	Phycoerythrin-Alexa 610, Ms IgG2a, S3.5, Caltag Laboratories
**CD45RA**	Phycoerythrin-Cyanine 5, Ms IgG2b,_k_, HI100, BD Biosciences
**CD25**	Phycoerythrin-Cyanine 7, Ms IgG_1,k_, M-A251, BD Biosciences
**CD31**	Fluorescein isothiocyanate, Ms IgG_1_,_k_, WM59, BD Biosciences
**FOXP3**	Phycoerythrin, Ms IgG_1,k_, 236A/E7, E-Bioscience

FACS acquisition was performed immediately with a BD LSR II cytometer and analyzed with BD FACSDiva software (BD Biosciences, San Jose, CA, USA) and FlowJo (Tree Star, Ashland, OR, USA). Flow cytometry performed in both fresh CBMCs and thawed CBMCs to ensure that the thawing process did not affect surface and intranuclear staining (**Figure S1**).

### Plasma separation, LPS quantification, and C-Reactive Protein (CRP) measurement

CB samples were centrifuged twice to separate plasma, which was stored at −80°C for batch analysis. Plasma was diluted to 10% or 20% with endotoxin free water and then heated to 85°C for 15 minutes to denature plasma proteins. Plasma levels of LPS were measured using a commercially available kit (Limulus Amebocyte Lysate [LAL] assay QCL-1000, Lonza, Walkersville, MD) according to the manufacturer's protocol. The lower level of detection for the assay was 0.7 pg/ml. Plasma levels of CRP were measured using a commercially available sandwich enzyme immunoassay kit and following manufacturer's protocol (Quantikine ELISA DCRP00, R&D, Minneapolis, MN). The minimum level of detection was 0.01 ng/ml.

### Chorioamnionitis determination and placenta examination

Placental examination is routinely performed for all preterm deliveries at TGH and is used for histological chorioamnionitis diagnosis. In most cases, histological chorioamnionitis is accompanied by evidence of invasion of pathogens in normally sterile tissues [15]. Histological chorioamnionitis was classified into one of three stages: Stage 1- neutrophils in placental chorionic plate only; stage 2- neutrophils throughout chorionic plate and sub amniotic connective tissue; and stage 3-necrotizing inflammation or multifocal abscess.

### Statistical analyses

Nominal variables are reported as frequencies, and continuous variables are reported as means ± standard deviations (SD) (parametric data), or as medians (25th–75th percentiles) (non-parametric data). Normality testing was performed using the Kolmogorov-Smirnov test. Data were analyzed by using SPSS statistical software (IBM SPSS for Windows, version 22, Armonk, NY) and GraphPad Prism 5 (GraphPad Software, La Jolla, CA, USA). Nominal variables were compared using chi-square analysis or Fischer's exact test as appropriate. Group medians were compared using a Mann- Whitney U test or Kruskal- Wallis test. Group means were compared using an independent samples t-test or ANOVA. Spearman rank or Pearson's correlation tests were used to measure the strength of association between variables. A p value ≤0.05 was considered statistically significant. Linear regression models were created in order to identify independent variables that predict CB CD31^−^ Tregs subpopulation percentages. The first model used gestational age as predictor. A second model included gestational age and CB LPS. A third model included aforementioned variables and histological chorioamnionitis.

## Results

### Patient characteristics

Seventy-one CB samples were obtained; 40 preterm and 31 term. The maternal characteristics are shown in **Table 2**. There were no statistically significant differences between preterm and term infant groups for maternal age, gravidity, parity, gender, race, mode of delivery, and history of maternal asthma or presence of histological chorioamnionitis. There was a statistical difference between term and preterm infants for gestational age (p<0.001) and birth weight (p<0.001). The use of prenatal antibiotics (71%, p<0.001), steroids (65%, p<0.001) and magnesium sulfate (50%, p<0.001) was more common in the preterm than the term group, as was maternal history of smoking (35%, p = 0.03).

**Table 2:** Maternal and Infant Characteristics.

	Term (n = 31)	Preterm (n = 40)	p value
**Maternal age (years: mean ± SD)**	31±7	29±6	0.53
**Gravidity (median (IQR))**	3 (2–4)	3 (1–4)	0.32
**Parity (median (IQR))**	1 (0–2)	2 (0–3)	0.44
**Gestational age (weeks; mean ± SD)**	39±1	31±3	<0.001
**Birth weight (grams; mean ± SD)**	3486±507	1730±667	<0.001
**Gender (% male)**	48.3	40	0.48
**Race (%)**			0.53
African American	13.8	23.1	
Caucasian	44.8	46.2	
Hispanic	41.4	30.7	
**Mode of delivery (%)**			0.06
Vaginal	19.3	40	
C-section	80.7	60	
**Prenatal use antibiotics (%)**	22.6	70.7	<0.001
**Prenatal use steroids (%)**	0	65	<0.001
**Prenatal use magnesium sulfate (%)**	0	50	<0.001
**APGAR score (1 minute)**			0.02
Below 5 (%)	0	15	
Above 5 (%)	100	75	
**Smoking (% yes)**	12.9	35	0.03
**Maternal asthma (% yes)**	9.7	7.5	0.74
**Histological chorioamnionitis (%)**			0.15
None	83.2	53.6	
Stage 1	5.6	3.6	
Stage 2	5.6	17.8	
Stage 3	5.6	25	

### Treg percentages and gestational age

**Figure 1:**
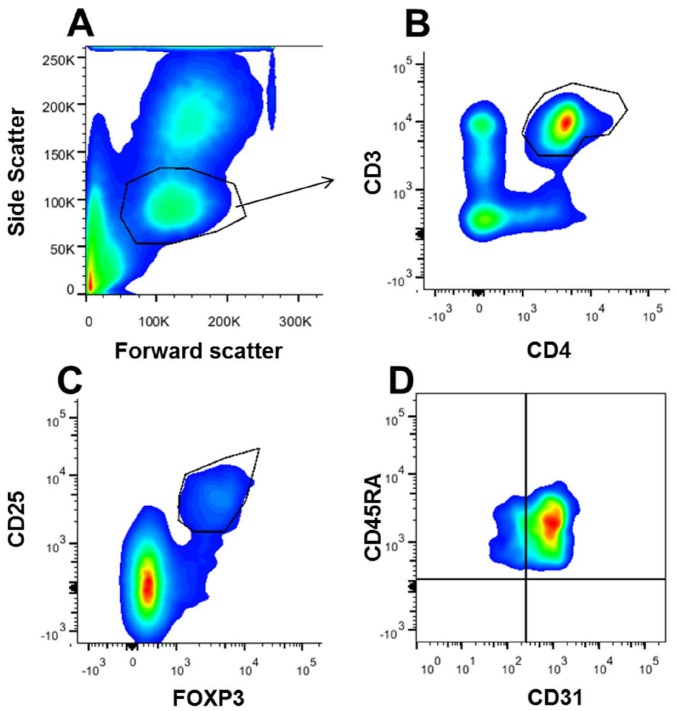
Cord blood flow cytometry analysis of regulatory T cells. Cord blood mononuclear cells were surface stained with fluorochrome labeled monoclonal antibodies followed by intranuclear staining for FOXP3, fixation and flow cytometric analysis. (**A**) Box-plot with lymphocytes gated based on size (FSC) and granularity (SSC). (**B**) Neonatal CD4^+^ T cells are selected based on CD3 and CD4 co-expression (box-plot). (**C**) Neonatal regulatory T cells (CD3^+^CD4^+^) are selected based on CD25 and FOXP3 co-expression (box-plot). (**D**) Regulatory T cells (CD3^+^CD4^+^CD25^+^FOXP3^+^) subpopulations selected by the expression of CD45RA and CD31 (CD45RA^+^CD31^+^ recent thymic emigrant, CD45RA^+^ CD31^−^ peripherally induced naïve regulatory T cell) (box-plot).

**Figure 2:**
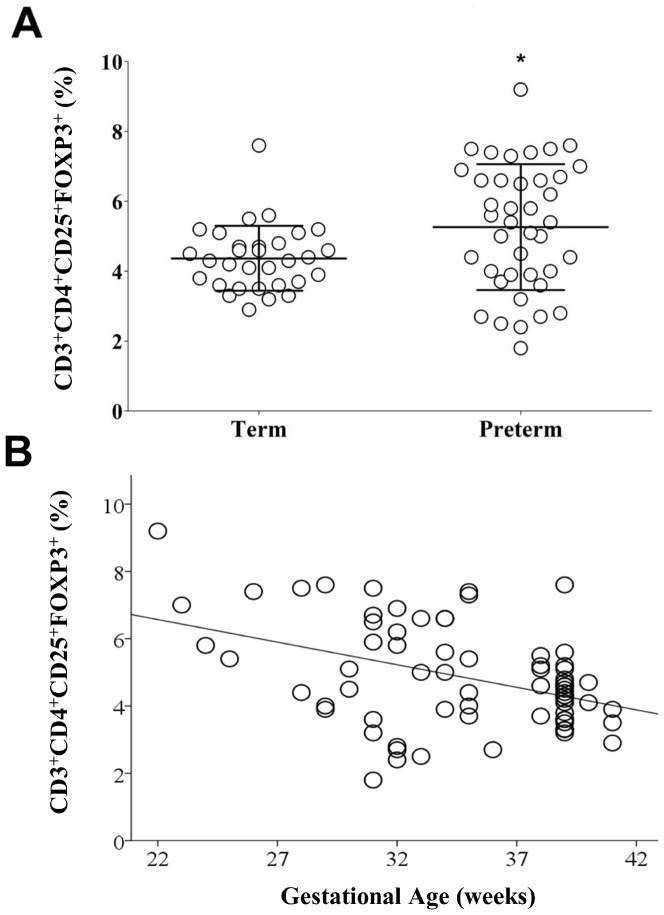
Increased percentage of Tregs in CB of preterm infants and correlates with gestational age. (**A**) Vertical scatter plot comparing mean percentage of Tregs (CD3^+^CD4^+^CD25^+^FOXP3^+^) in CB of term (n = 31) and preterm (n = 40) infants. Statistical significance was determined by independent sample T-test (*p = 0.015) (**B**) Scatter plot graph showing a negative correlation between gestational age (X-axis, in weeks) and CB Tregs percentage (Y-axis). Pearson's correlation test used (r = −0.414, p = 0.0001)

Tregs were identified by size, granularity (**Figure 1A**), and co-expression of CD3 and CD4 (**Figure 1B**), CD25 and FOXP3 (**Figure 1C**) and CD45RA (**Figure 1D**). The percentage of Tregs (CD3^+^CD4^+^CD25^+^FOXP3^+^) was higher in preterm (mean ± standard deviation (SD)  = 5.3±1.8%) vs. term CB (4.3±0.9%) (p = 0.015) (**Figure 2A**). There was a significant negative correlation between the percentage of Tregs and gestational age (Pearson's r = −0.414, p = 0.0001) (**Figure 2B**).

### Treg subpopulations in term and preterm infants

**Figure 3:**
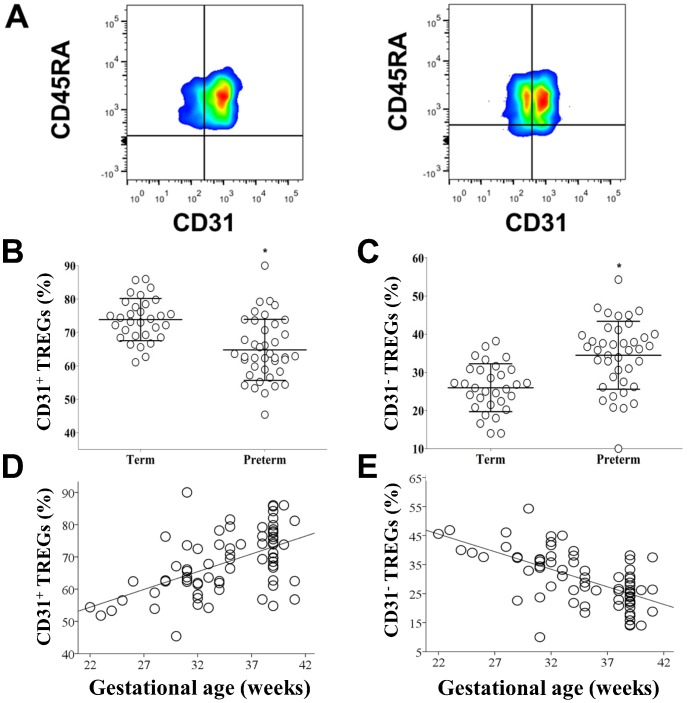
Tregs subpopulations percentages are different between term and preterm infants. (**A**) Term CB (first box plot) and preterm CB (second box plot). Tregs were examined for expression of the surface markers CD45RA and CD31. (**B**) Vertical scatter plot comparing mean percentage of CD3^+^CD4^+^CD25^+^FOXP3^+^CD45RA^+^CD31^+^ in CB of term and preterm infants. Statistical significance was determined by independent sample T-test (*p = 0.0001). (**C**) Vertical scatter plot comparing median percentage of CD3^+^CD4^+^CD25^+^FOXP3^+^CD45RA^+^CD31^−^ in CB of term and preterm infants. Statistical significance was determined by independent sample T-test (*p = 0.0001). (**D**) Scatter plot graph showing a positive correlation between gestational age (X-axis, in weeks) and CB CD31^+^ Tregs percentage (Y-axis). Pearson's correlation test used (r =  0.54, p = 0.0001). (**E**) Scatter plot graph showing a positive correlation between gestational age (X-axis, in weeks) and CB CD31^−^ Treg percentage (Y-axis). Pearson's correlation test used (r = −0.62, p = 0.0001).

Tregs subpopulations were identified by the surface expression of CD45RA and CD31. RTE Tregs were defined by the surface expression of CD45RA and CD31. Inducible Tregs (or antigen driven Tregs) were defined by the presence of CD45RA and absence of CD31. The percentage of CD31^+^ Tregs (**Figure 3A**) was significantly higher in term than preterm CB (mean ± SD 73±8.8% vs. 62.8%, p = 0.0001) (**Figure 3B**), while the percentage of CD31^−^ Tregs was significantly higher in preterm than term CB (34.5±8.9% vs. 24.7±6.6%, p = 0.0001) (**Figure 3C**). There was a significant correlation between the percentage of both subpopulations of Tregs (CD31^+^ and CD31^−^) and gestational age (CD31^+^ Pearson's r = 0.54, p = 0.0001 and CD31^−^ Pearson's r = −0.62, p = 0.0001) (**Figure 3D and 3E**) Differences in CD31 subpopulations were only observed in the Tregs subgroup and not in the total CD4+ lymphocyte group (**Figure S2**).

### Factors associated with treg homeostasis

**Figure 4:**
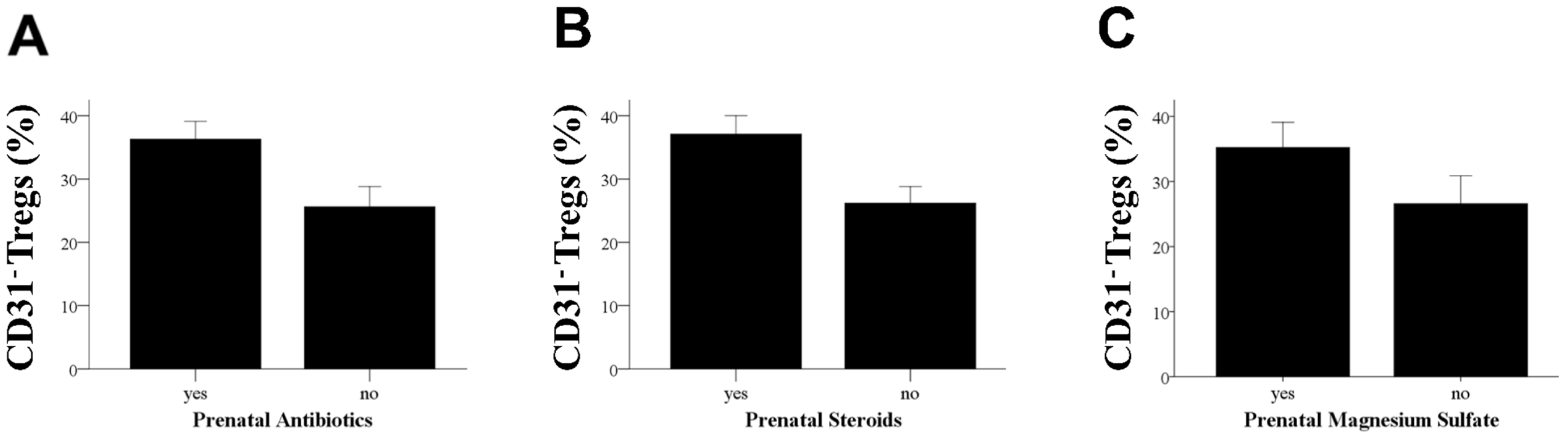
Maternal factors affect the percentages of Treg phenotypes in CB. (**A**) Column bar graph comparing median and confidence intervals of the percentage of CD31^−^ Tregs in infants (both term and preterm) whose mothers had received prenatal antibiotics. “Yes” indicates mother received prenatal antibiotics and “No” indicates no antibiotics were given. Statistical significance was determined by Mann-Whitney U test (*p<0.0001). (**B**) Column bar graph comparing median and confidence intervals of the percentage of CD31^−^ Tregs in infants (both term and preterm) whose mothers had received prenatal steroids. “Yes” indicates mother received prenatal steroids and “No” indicates no steroids were given. Statistical significance was determined by Mann-Whitney U test (*p<0.0001). (**C**) Column bar graph comparing median and confidence intervals of the percentage of CD31^−^ Tregs in infants (both term and preterm) whose mothers had received prenatal magnesium sulfate. “Yes” indicates mother received prenatal magnesium sulfate and “No” indicates no magnesium sulfate was given. Statistical significance was determined by Mann-Whitney U test (* p = 0.005).

There was no significant difference in the percentage of CB Tregs, CD31^+^Tregs or CD31^−^ Tregs in both term and preterm based on sex, race, 1 minute- APGAR scores, delivery mode, or maternal smoking. There was a significantly higher percentage of CD31^−^ Tregs in CB from infants whose mothers had received prenatal antibiotics (36.3% vs. 25.6%, p<0.0001) (**Figure 4A**), steroids (37.1% vs. 26.2%, p<0.0001) (**Figure 4B**) or magnesium sulfate (34.5% vs. 26.7%, p = 0.005) (**Figure 4C**).

**Figure 5:**
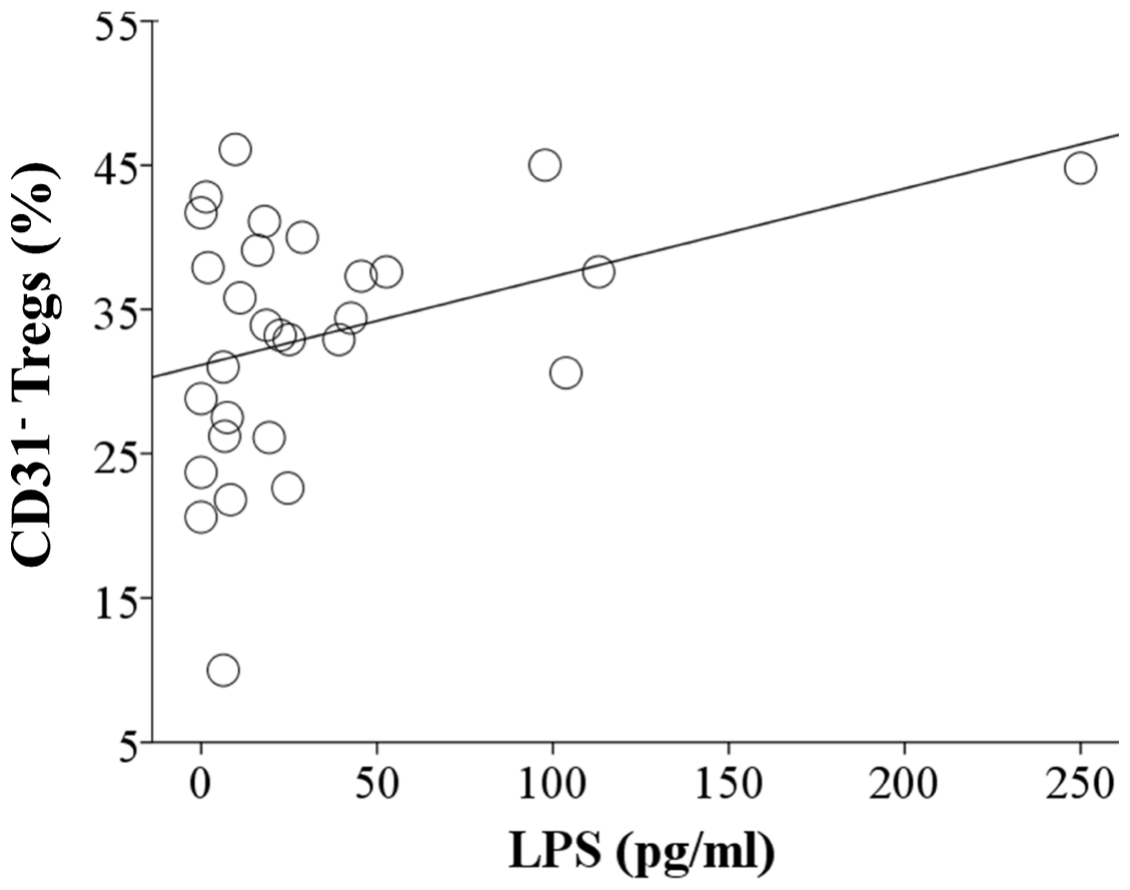
CB LPS correlates with the percentage of CD31^−^ Tregs of preterm infants. Scatter plot graph showing correlation between CB LPS levels (X-axis) and CD31^−^ Tregs (Y-axis, percentage). Spearman rank correlation test used (r = 0.373, p = 0.046).

**Figure 6:**
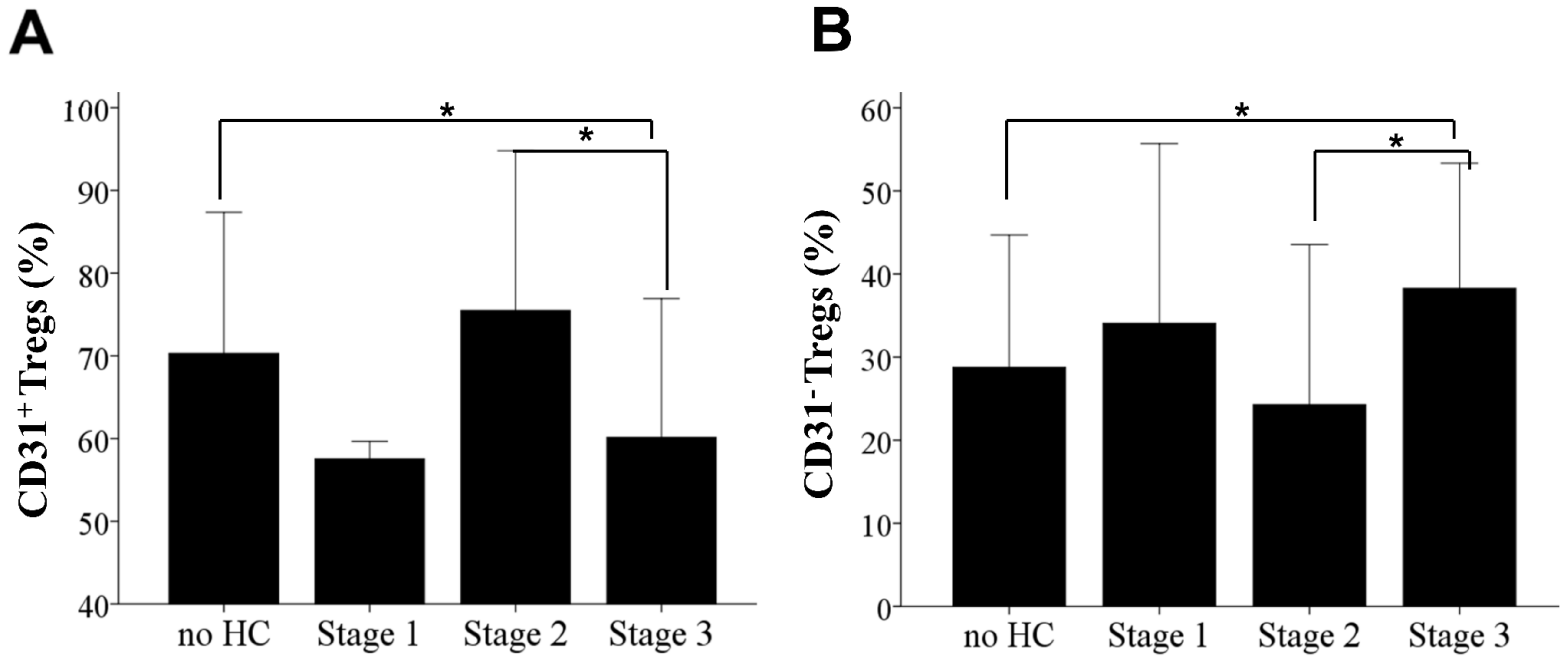
Histological Chorioamnionitis and Tregs Homeostasis. (**A**) Column bar graph representing means and standard deviations of CD31^+^ Treg percentage in CB with different staging of histological chorioamnionitis and no chorioamnionitis. Statistical significance between all groups (p = 0.018) was determined by one way ANOVA with Bonferroni post hoc test. (**B**) Column bar graph representing means and standard deviations of CD31^−^ Treg percentage in CB with different staging of histological chorioamnionitis and no chorioamnionitis. Statistical significance between groups (p = 0.013) was determined by one way ANOVA with Bonferroni post hoc test.

The state of in utero inflammation was assessed by measuring CB plasma CRP, plasma LPS and by placental pathology in both term and preterm infants. There was no significant correlation between CB CRP and LPS levels and the percentage of Tregs (combined term and preterm) (CRP r = 0.137, p = 0.439; LPS r = 0.48, p = 0.758), CD31^+^ Tregs (CRP r = −0.143, p = 0.420; LPS r = −0.071, p = 0.653) and CD31^−^ Tregs (CRP r = 0.168, p = 0.342; LPS r = 0.81, p = 0.604). Nevertheless, for the preterm CB group only, we found a positive correlation between the percentage of CD31^−^ Tregs and CB LPS (r = 0.373, p = 0.046) (**Figure 5**). In addition, preterm infants with stage 3 chorioamnionitis compared to no chorioamnionitis had a significantly lower percentage of CD31^+^ Tregs (60.15±8.4% vs. 70.3±8.5%, p = 0.028) (**Figure 6A**) and a significantly higher percentage of CD31^−^ Tregs (38.3±7.5% vs. 28.8±7.9%, p = 0.033) (**Figure 6B**). No differences between CD31 subpopulations were seen between no chorioamnionitis and either histological chorioamnionitis stage 1 or stage 2. A significant difference was seen for both CD31^+^ and CD31^−^ Tregs between stage 2 and stage 3 chorioamnionitis (p = 0.011 and p = 0.017, respectively).

### Predictors of CB Treg subpopulations

To control for multicollinearity, birth weight was not included in the prediction models since it was highly correlated with gestational age (Pearson's r = 0.892, p<0.0001). The prediction model (**Table 3**) with gestational age alone offered the strongest predictive value (R^2^ = 0.381). The variable of gestational age alone was higher than the combination of all clinical variables (R^2^ = 0.349). The clinical variables of prenatal use of steroids (p = 0.786), antibiotics (p = 0.059) and magnesium sulfate (p = 0.589) showed no significant predictive values for CD31^−^ Tregs percentages when corrected for gestational age.

**Table 3:** Outcome of predictive model for Treg populations.

Predictors	Model 1	Model 2	Model 3
Gestational age	+	+	+
Cord blood LPS		+	+
Histological Chorioamnionitis			+
***R***	0.617	0.559	0.591
***R^2^***	0.381	0.313	0.349
***P*** ** value**	<0.0001	0.001	0.002

## Discussion

We report a gestational age-dependent difference in the percentage of total Tregs, in which preterm infants of lower gestational ages have an increased percentage of Tregs; there were no other maternal or fetal factors associated the percentage of total Tregs. In addition, we report the presence of two populations of Tregs (CD31^+^ and CD31^−^) in CB of term and preterm infants, and the percentages of these populations are associated with different maternal and fetal characteristics. Factors associated with differences in the percentage of CD31^−^ Tregs included the use of prenatal antibiotics, steroids and magnesium sulfate. In addition, the percentage of CD31^−^ Tregs was significantly higher in CB of preterm pregnancies associate with inflammation and prenatal LPS exposure.

To our knowledge, this study is the first to described two different populations of Tregs (RTEs and peripherally induced Tregs) in term and preterm infants. These two populations have been extensively studied in animal models [16], [17] and adults [4], [18], [19] but they are not well described in neonates.

Our study describes the composition of the Treg pool shifting towards peripherally induced Tregs during prematurity and prenatal inflammation similar to the profile seen with aging. This may be due to decreased thymic output of Tregs. Our findings suggest that a shift in the homeostatic composition of Treg subsets related to reduced de novo generation of RTE Treg cells may contribute to prematurity (lower gestational age) and highlights the importance of this particular population of Tregs in maintaining maternal fetal tolerance. It is also interesting that the differences in Tregs subpopulations are limited to events with higher levels of inflammation. These higher levels of inflammation are represented by increased levels of LPS and higher staging of chorioamnionitis (stages 2 and 3). Other factors may also play a role in the shift towards peripherally induced Tregs during prematurity. These factors include cytokines and other bacterial products.

There are several limitations of our study. First, our CB analysis captures only one timepoint, providing a snapshot of in utero influences on the Treg pool. A longitudinal study of the Treg pool of term and neonates infants would be of great interest, and would enable us to investigate the relationship between the Treg pool and clinical outcomes. Also, a larger preterm sample size is needed to more thoroughly investigate the relationship between cell populations and clinical variables. Our study does not have enough power to make any conclusions in terms of Tregs populations and clinical outcomes. In addition, the association between the percentage of CD31^−^ Tregs and the use of prenatal antibiotics, steroids or magnesium sulfate could be misleading, because these factors are more predominant in the preterm group, thus, the differences could be related to gestational age differences as demonstrated by our predictive models. In addition, new cell surface markers have been used to identify Tregs in blood. CD127 (IL-7 receptor) has been shown to be low in Tregs and provides a flexible alternative to the transcription factor FOXP3 [20], [21]. Our samples were not stained for CD127 but stained for FOXP3 (intranuclear). CD127 is a surrogate marker for Tregs and intranuclear staining with FOXP3 is more specific.

The results of the study have implications for the understanding of neonatal immune regulation since Tregs are crucial for immune tolerance and autoimmunity. Our group previously described altered T lymphocyte cell populations in preterm infants. We speculate that in utero inflammation, chorioamnionitis and LPS can further alter the Treg pool homeostasis of preterm infants. Early exposure to antigens and cytokines can alter the proportion of RTEs and naïve induced Tregs in the Treg pool. The clinical implications of these changes in the Treg pool are not clear, and it is not known if the induced Tregs anti-inflammatory properties are altered. Future directions include investigating the relationship between changes in Treg pool homeostasis and clinical outcomes such as development of necrotizing enterocolitis, bronchopulmonary dysplasia and asthma in preterm infants.

In conclusion, the peripheral Treg pool of preterm infants may be altered by lower gestational age, prenatal exposure to inflammation and chorioamnionitis; however, the clinical implications of this finding are not yet understood.

## Supporting Information

Figure S1 Surface and intranuclear staining for Tregs in fresh cord blood mononuclear cells (CBMCs) and thawed CBMCs. CBMCs were surface stained with fluorochrome labeled monoclonal antibodies followed by intranuclear staining for FOXP3, fixation and flow cytometric analysis.
(TIFF)

Figure S2 Term CB (first column) and preterm CB (second column). CD4+ Lymphocytes and Tregs were examined for expression of the surface marker CD31.
(TIFF)
