# Supplementary figures and images for: Alterations in Regulatory T Cell Subpopulations Seen in Preterm Infants
Source: PLoS One. 2014 May 5;9(5):e95867. doi: 10.1371/journal.pone.0095867 (PMC4010410; doi:10.1371/journal.pone.0095867)

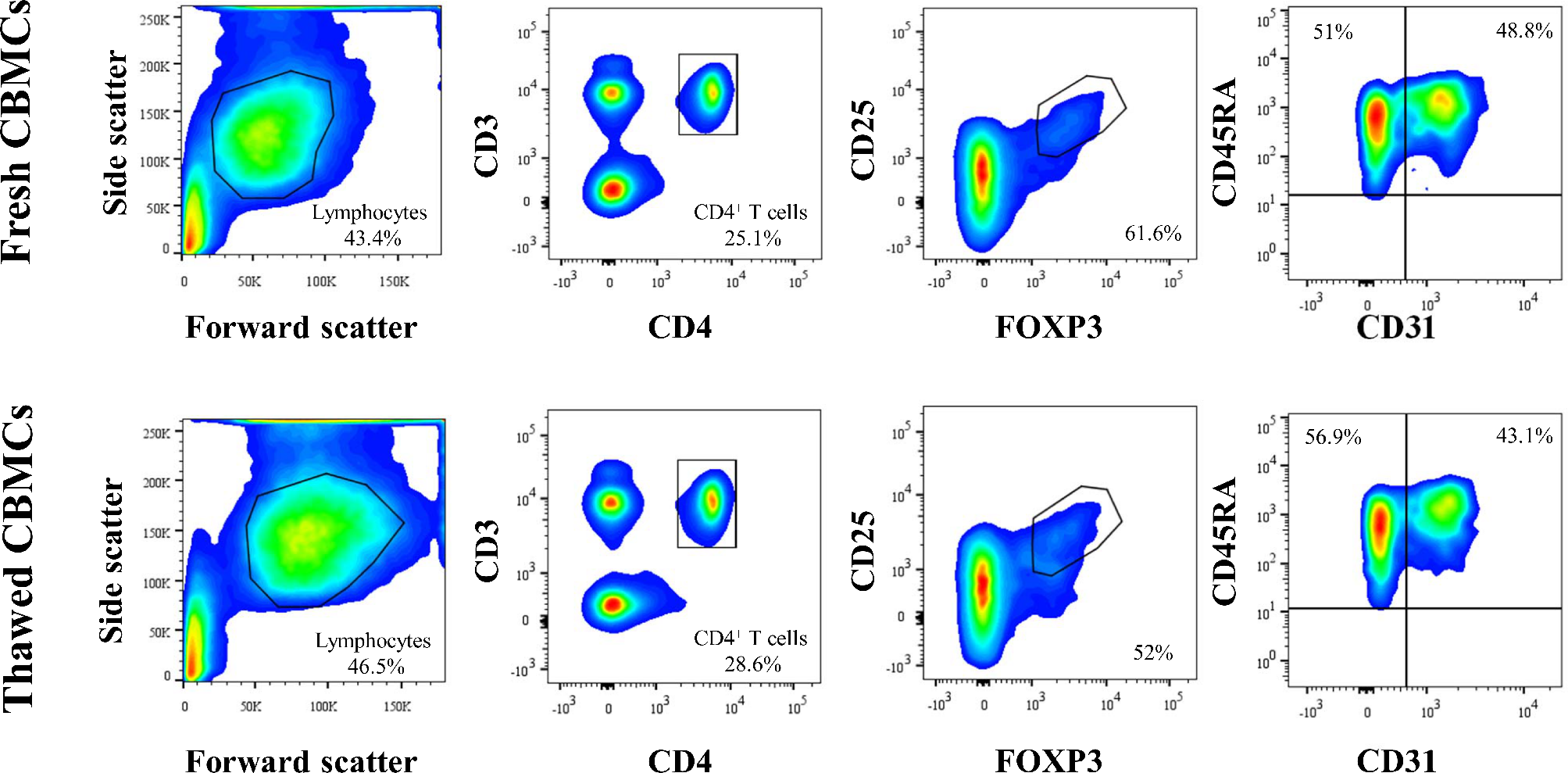

Supplement: Figure S1 — Surface and intranuclear staining for Tregs in fresh cord blood mononuclear cells (CBMCs) and thawed CBMCs. CBMCs were surface stained with fluorochrome labeled monoclonal antibodies followed by intranuclear staining for FOXP3, fixation and flow cytometric analysis. (TIFF) [file pone.0095867.s001.tif]

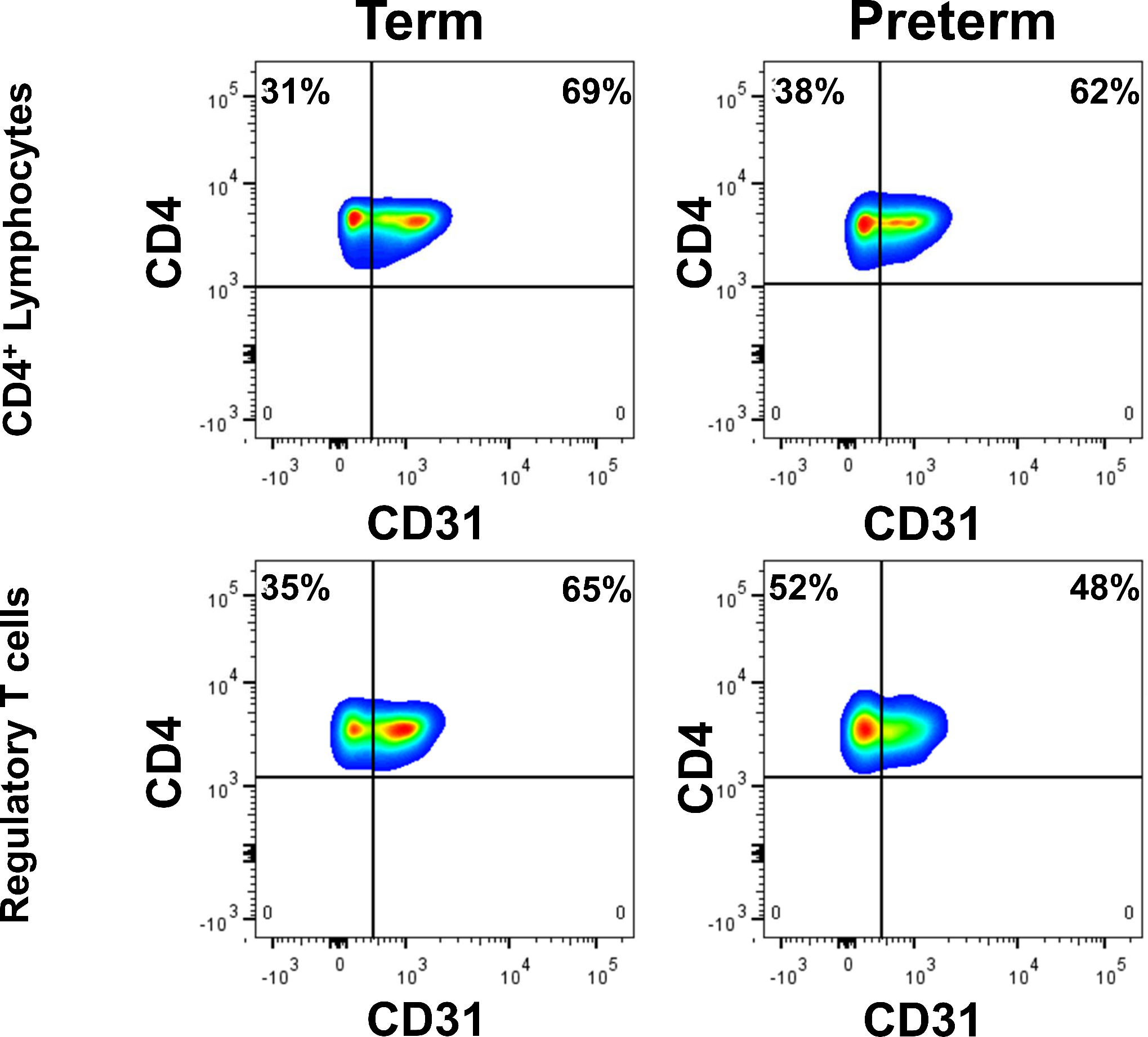

Supplement: Figure S2 — Term CB (first column) and preterm CB (second column). CD4+ Lymphocytes and Tregs were examined for expression of the surface marker CD31. (TIFF) [file pone.0095867.s002.tif]
